# Integrated Whole Genome and Transcriptome Sequencing as a Framework for Pediatric and Adolescent AML Diagnosis and Risk Assessment

**DOI:** 10.21203/rs.3.rs-5775959/v1

**Published:** 2025-01-22

**Authors:** Lu Wang, Rebecca Voss, Victor Pastor, Maria Cardenas, Priyadarshini Kumar, Jamie Maciaszek, Maria Namwanje, Jing Ma, Jennifer Neary, Meiling Jin, Masayuki Umeda, Mark Wilkinson, Debbie Payne-Turner, Mohammad Eldomery, Jingqun Ma, Jiali Gu, James Dalton, Samantha Melton, Yen-Chun Liu, Scott Foy, Michael Rusch, David Wheeler, Jinghui Zhang, Kim Nichols, Seth Karol, Hiroto Inaba, Raul Ribeiro, Jeffrey Rubnitz, Jeffery Klco

**Affiliations:** St Jude Children’s Research Hospital; St. Jude Children’s Research Hospital; St. Jude Children’s Research Hospital; St. Jude Children’s Research Hospital; St. Jude Children’s Research Hospital; St Jude Children’s Research Hospital; St. Jude Children’s Research Hospital; St. Jude Children’s Research Hospital; St. Jude Children’s Research Hospital; St. Jude Children’s Research Hospital; St. Jude Children’s Research Hospital; St. Jude Children’s Research Hospital; St. Jude Children’s Research Hospital; St. Jude Children’s Research Hospital; St. Jude Children’s Research Hospital; St Jude Children’s Research Hospital; St. Jude Children’s Research Hospital; St. Jude Children’s Research Hospital; St. Jude Children’s Research Hospital; St. Jude Children’s Research Hospital; St. Jude Children’s Research Hospital; St. Jude Children’s Research Hospital; St. Jude Children’s Research Hospital; St. Jude Children’s Research Hospital; St. Jude Children’s Research Hospital; St. Jude Children’s Research Hospital; St. Jude Children’s Hospital; St. Jude Children’s Research Hospital; St. Jude Children’s Research Hospital

**Keywords:** Whole genome sequencing, Whole transcriptome sequencing, AML

## Abstract

Pediatric acute myeloid leukemia (AML) exhibits distinct genetic characteristics, including unique driver alterations and mutations with prognostic and therapeutic significance. Emerging rare, recurrent genetic abnormalities and their associations with outcomes emphasize the need for high-throughput molecular diagnostic tools. Whole genome sequencing (WGS) reliably detects key AML biomarkers such as structural variants, mutations, and copy number alterations. Whole transcriptome sequencing (WTS) complements WGS by uncovering oncogene expression patterns, allele-specific expression, and gene expression signatures. In this study, we describe an integrated WGS and WTS clinical workflow for routine pediatric AML diagnosis and present a systematic evaluation of its application compared to conventional cytogenetics and standard molecular diagnostic methods. Our findings demonstrate that the integrated WGS and WTS (iWGS-WTS) approach improves the identification of clinically relevant genetic alterations, enhancing precise disease classification and risk assessment. Moreover, with advancements in workflow and bioinformatics pipelines, the testing turnaround time can be optimized to meet the demands of clinical decision-making, positioning iWGS-WTS as a practical and superior alternative to traditional diagnostic methods in pediatric AML management.

## Introduction

Acute myeloid leukemia (AML) comprises a heterogeneous group of genetically distinct disorders. Over the past decade, next-generation sequencing (NGS) has uncovered the molecular landscape of AML, highlighting recurrent genetic alterations and their interactions (1–4). These insights, combined with efforts to understand myeloid cell transformation, have shaped molecular classification of AML. The updated WHO classification retains many previously defined subtypes and includes additional genetically related entities (5). Although AML accounts for only 20% of pediatric acute leukemia cases, it is the leading cause of childhood leukemia mortality (6). Pediatric AML shares features with adult AML but also exhibits distinctive genetic characteristics (3, 7). Clinical tests for common genomic alterations are widely used to stratify patients and guide treatment. However, the continued discovery of rare but recurrent genetic abnormalities in acute leukemia and the recognition of their association with patient outcomes underscores the need for high-throughput molecular diagnostics and improved testing strategies for unbiased detection of genetic abnormalities.

Although the potential clinical utility of whole genome sequencing (WGS) and whole transcriptome sequencing (WTS) in oncology has been demonstrated, its routine adoption in cancer diagnostics remains limited worldwide. Comprehensive clinical genomics testing for leukemia molecular diagnosis, combining WGS/whole exome sequencing (WES) and WTS was implemented at St. Jude Children’s Research Hospital (SJCRH) in 2016. Here, we present the first systematic evaluation of our real-time clinical genomic testing experience and compare our strategy with traditional AML molecular diagnostics, demonstrating how an integrated WGS and WTS (iWGS-WTS) approach can serve as a primary diagnostic tool in pediatric AML care by identifying clinically relevant genetic and genomic alterations.

## Results

### Characteristics of patients and samples

Of the 154 patients in this study cohort, 75 were male and 79 were female, and the median age at diagnosis was 11 years old (1 week – 22 years) (Supplementary Table S1). Samples from the first available timepoint were analyzed, comprising 13 treatment-related AML (tAML), 27 relapsed AML cases of which the initial diagnosis and treatment were conducted outside SJCRH, and 114 cases with de novo AML. The median bone marrow blast count was 66% (5–96%). The percentage of blasts in the samples studied was ≥ 20% except for four cases: two patients with newly diagnosed Down Syndrome (DS)-related AML (5% and 13% blasts, respectively), one patient was initially diagnosed with AML with myelodysplasia-related changes of whom only a post-treatment sample with 9% blasts was available for testing, and one patient with relapsed AML with *NPM1* mutation (15% blasts).

### Technical considerations

#### Sequencing QC metrics

Across this cohort, we achieved a median WGS depth of 61x (range: 39x – 149x), with a median total read count of 1.67 billion reads, a median mapping rate of 94.7% and a duplication rate of 7.3% (Supplementary Figure S2). WGS QC pass was determined based on the percentage of exons with an average coverage above 45x, a percentage below 40% indicating suboptimal WGS QC. Median percentage of exons covered at > 45x was 86.9%, only 6 cases had less than 40% of exons (range: 23% ~ 38%) received sequencing coverage > 45x. All 6 cases proceeded with further analysis. As for WTS, the median total read count was 161 million reads, with a median mapping rate of 98.4% and a median duplication rate of 32.7% (Supplementary Figure S3 A-B). Additionally, we assessed overall protein-coding gene coverage in WTS. Overall, ~ 92% of cases showed at least 12,000 coding genes with ≥1x sequencing coverage when total mapped reads were ≥ 50 M (Supplementary Figure S3C). WES was performed on all tumor samples at a median sequencing depth of 137x. A subset of patients (n = 39) underwent targeted ‘Myeloid Panel’ NGS with an overall median sequencing coverage of 1453x (range 962x – 1922x).

#### Estimating WGS testing performance for the detection of single nucleotide variants (SNV), small insertion/deletion (Indel) and large-scale copy number variants using tumor cell line

We utilized the COLO829 tumor cell line to estimate the limit of detection for tumor content and variants at low variant allele frequency (VAF) by WGS. This cell line was diluted with its matched normal cell line COLO829BL to achieve a final tumor DNA content of 40%, 30% and 25%, respectively. Both the original cell line and the diluted cell line mixed sample were analyzed by WGS and WES. Twelve tumor-acquired variants (9 SNVs and 3 indels) identified in the original cell line with VAFs ranging from ~ 29% to ~ 70%, were selected for evaluation. In the mixed cell line sample with 25% tumor DNA content, these variants were expected at VAFs ranging from approximately 7–17.5%; indeed, WGS successfully detected all variants with high reproducibility in VAF estimate (overall inter- and intra-run standard deviation: 0.04). Also, the data demonstrated good correlation in variant detection and VAF estimates between WGS and WES (Supplementary Figure S4 A-C).

Large-scale (> 5 Mb) copy number variants (LS-CNV) of 1 or 2 copy gains of chromosomes 1q, 2, 3, 7, 12 and 19 were chosen for evaluation (8). In mixed cell line samples of 40% tumor DNA content, all LS-CNVs were detected. In contrast, in the mixed cell line sample of 30% tumor DNA, the anticipated LS-CNVs were shown as multiple segmental CNVs in the pipeline output, and only a portion met the diagnostic criteria. Consequently, the anticipated LS-CNVs could not be established accurately in the sample of 30% tumor DNA (Supplementary Figure S4D). When the tumor DNA content dropped to 25%, the expected CNVs appeared further fragmented in pipeline analysis and many segments did not meet diagnostic criteria. Overall, this pilot study indicated that low tumor purity has more impact on LS-CNV detection than the detection of SNVs/indels.

#### Comprehensive tumor genomic and transcriptomic profiling and comparison between NGS platforms Detection of SNVs/indels and comparison of iWGS-WTS to both WES and targeted NGS

Using the iWGS-WTS approach, a total of 333 pathogenic or likely pathogenic (P/LP) variants in 135/153 patients were considered valid for reporting (Supplementary Fig. 1A for variant calling criteria). Generally, a cut-off of 5% VAF at a minimum of 10 total reads in WGS was applied, while AML driver variants presenting at < 5% VAF were evaluated on a case-by-case basis. For example, two patients with Down Syndrome (SJ030077, 13% blasts; SJ031477, 5% blasts) showed *GATA1* variants at 4.8% and 3.1%, respectively. Another patient (SJ032424) with relapsed AML and a blast count of 15% exhibited *NPM1* mutation at a VAF of 2.8%, consistent with the mutation identified in the patient’s diagnostic sample. Notably, all the three variants with low VAF in WGS demonstrated VAFs exceeding 25% in WTS (Supplementary Table S2).

**Figure 1:**
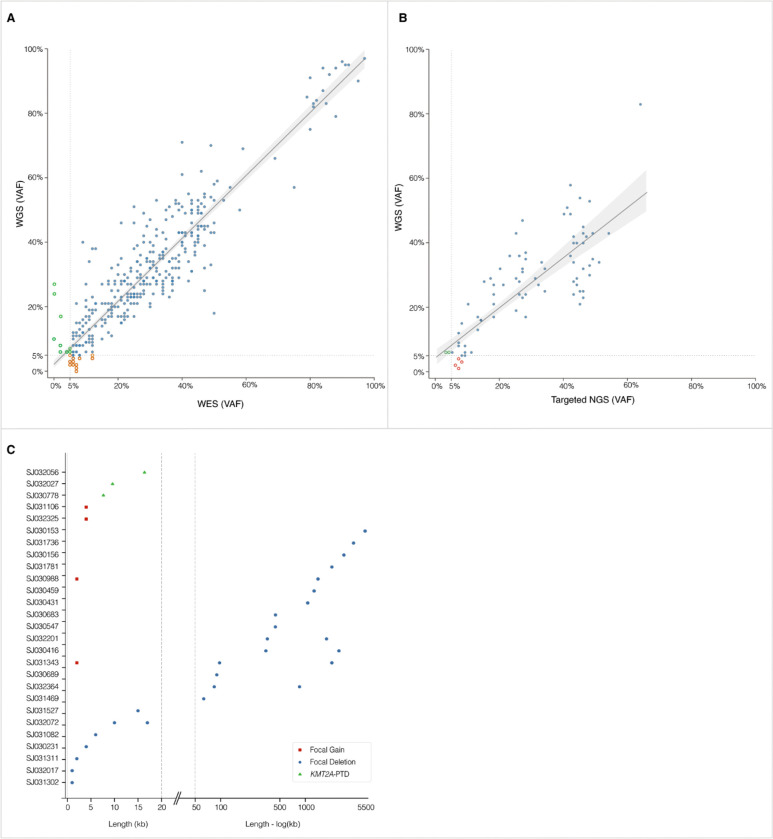
Detection of SNVs/indels and focal CNVs. **A)** Comparison of WGS and WES in the detection of SNVs and Indels. Variant allele frequency (VAF) for Whole Genome Sequencing (WGS) and Whole Exome Sequencing (WES) is shown on the x and y axes, respectively. Variants with less than 5% VAF are shown in green for WES and orange for WGS. High concordance in VAF estimates among the variants considered reportable by both WGS and WES observed: correlation coefficient (r) = 0.9 (slope = 0.88). **B)** Comparison of WGS and targeted panel NGS in the detection of SNV and Indel. VAF is shown for WGS and targeted Next Generation Sequencing (NGS) on the x and y axes, respectively. Variants with less than 5% VAF are shown in green (WES) and orange (WGS). Correlation of VAF estimates showed a correlation coefficient (r) of 0.83 (slope = 0.86). **C)** Focal CNVs detected by WGS are presented according to size (in kb) on the x-axis. Gains are red squares, deletions blue dots and partial tandem deletions (PTDs) green triangles.

We next compared the capability of WGS with WES in detecting SNVs/indels with VAF ≥ 5%. Of the 330 variants with VAF ≥ 5% called by WGS, 321 were detected by WES with VAF > 5%. Among the nine additional variants not called by WES, the majority were complex indels. Manual review of WES BAM files revealed 7/9 of those variants, with VAFs ranging from 0.3–4.9%. Conversely, 15 variants detected by WES with VAFs ranging from 5–12.5% (average 6.8%), were not called by WGS. Manual review of WGS BAM files showed all but one, at an average VAF of 3.1% (range 1.1–4.8%). Overall, among the 345 SNVs and small indels detected by WGS and/or WES in this cohort, 93% with VAF ≥ 5% (range: 5–97%) were considered valid to report by both platforms (Supplementary Table S2), with high concordance in VAF estimates between WGS and WES (r = 0.9, Fig. 1A).

Across the 39 cases tested by the targeted DNA sequencing (Myeloid Panel, 75 genes) as well, 74 P/LP variants with VAF ≥ 5% were obtained from the panel testing pipeline. Of these, 70 were reported by WGS, adhering to the established variant calling criteria. An additional variant was detected by WGS with VAF > 5%, and met all other reporting criteria, for which the targeted panel NGS testing showed a VAF of 4%. Five additional variants with estimated VAF between 3% and 5% reported by the targeted panel testing, all of them were observed in WGS BAM les upon manual review; however, they did not meet the established reporting criteria (Supplementary Table S2). Variants called by both WGS and the targeted NGS assay ranged from 5–83% VAF in WGS with high concordance in VAF estimates between the two sequencing platforms (r = 0.83, Fig. 1B).

#### Detection of *FLT3*-intragenic tandem duplications (ITD), focal and large scale CNVs

Among the 153 patients analyzed by WGS, 16 patients were found to harbor a total of 28 *FLT3*-ITDs. Seventeen of 28 *FLT3*-ITDs were detected by both WGS and WTS; 10 showed strong evidence in WTS but weak or no evidence in WGS and were thus considered borderline findings; the remaining one exhibited weak evidence in both WGS and WTS. All cases underwent PCR-based fragment analysis, which confirmed all 28 *FLT3*-ITD variants (Supplementary Table S3A). In addition, PCR identified one subclonal *FLT3*-ITD (ratio of *FLT3*-ITD to wild-type: 0.02) in SJ030286 that was not detected by WTS or WGS.

Furthermore, WGS revealed 42 P/LP focal CNVs (< 5Mb) in 24 patients, including 15 alterations smaller than 50 kb (12 ≤ 10 kb), presenting as intragenic/exonic CNVs leading to truncation of the functional protein (Fig. 1C, Supplementary Table S3B). Among the alterations smaller than 50 kb, the recurrent findings were deletions in *CBL* (n = 4) affecting exons 8 and/or 9 and *KMT2A* partial tandem duplication (*KMT2A*-PTD, n = 3). All intragenic CNVs were deemed valid when two types of supporting evidence derived from WGS, i.e., read-depth and corresponding soft-clipped reads, were present (Supplementary Figure S5). All *KMT2A*-PTD alterations were also supported by WTS, which identified in-frame fusions of exons 7 or 8 upstream of a 5’ exon (exons 2, 3, or 4).

Genome-wide large-scale (> 5 Mb) copy number variants (LS-CNV) detected by WGS analysis are presented in Supplementary Table S4. In general, 163 LS-CNV events were identified in 83 patients at a median of one event per patient (range: 1–13). More details to follow.

#### Detection of AML-associated chromosome rearrangements / gene fusions and comparison of WGS and WTS

**Figure 2:**
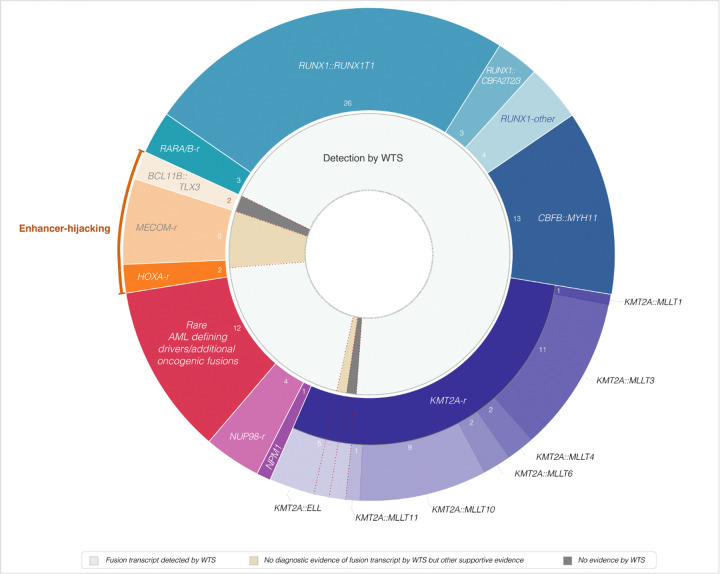
Comparison of WGS and WTS in the detection of AML-associated oncogenic gene fusions/chromosomal translocations. **Outer ring:** Oncogenic/likely oncogenic fusions detected by WGS. Enhancer hijacking fusions are marked. The remaining events are predicted to form chimeric fusion transcripts. **Inner ring:** Fusion detection by WTS. Events missed by WTS are indicated in gray, those that did not show diagnostic evidence of a fusion transcript by WTS but were confirmed by other supportive evidence are shown in beige.

WGS analysis for structural variants suggested 107 AML-associated oncogenic or likely oncogenic fusions in 106 cases (SJ031259 harbored both *CBFB::MYH11* and a *CNTRL::KIT* fusion), of which 97 were predicted to produce fusion oncogenes/chimeric oncoproteins and 10 were suspected to be enhancer-hijacking structural alterations that activate adjacent oncogenes (Fig. 2). WTS analysis alone diagnosed 95/97 (98%) P/LP WGS-detected fusion oncogenes, with no false positive findings (Supplementary Table S5) according to our established diagnostic criteria. In the remaining two cases, WGS suggested *KMT2A::ELL* fusions: (1) SJ031554, *KMT2A* intron 11 fused to a region at 19p13.3 that is ~ 3.4 kb upstream of *ELL*; and (2) SJ031359, *KMT2A* exon 9 fused to *ELL* intron 8. In SJ031554, although WTS did not provide sufficient diagnostic evidence of a *KMT2A::ELL* fusion, manual review of the WTS BAM le revealed a single read supporting an in-frame fusion transcript of *KMT2A* exon 11 fused to *ELL* exon 2; whereas in WTS data of SJ031359, no read for a chimeric transcript of *KMT2A::ELL* was observed. Both cases were subjected to targeted RNA sequencing (Archer^™^ FUSION*Plex*^™^ Pan-Heme panel, IDT, USA), which confirmed the chimeric transcript with the fusion junction between *KMT2A* exon 11 and *ELL* exon 2 in SJ031554 and revealed an in-frame fusion transcript with *KMT2A* exon 9 fused to a cryptic exon from *ELL* intron 8 then to *ELL* exon 9 in SJ031359 (Supplementary Figure S6).

The 10 cases with suspected enhancer hijacking fusions detected by WGS were predicted to dysregulate *MECOM* in six cases, *HOXA* genes in two (SJ030410 and SJ032072), and *BCL11B* (or *TLX3*) in two (SJ030153 and SJ030431) based on WGS analysis. Among them, only one case had a chimeric fusion transcript revealed by WTS: a fusion transcript for *HOXA10::PLEK* in SJ032072 along with overexpression of *HOXA10* (Supplementary Fig. 7C-D). Across the six *MECOM*-rearranged cases, WGS showed breakpoints on 3q26.2, ranging from 3.8 kb – 300 kb upstream and from 78 kb – 146 kb downstream of *MECOM* (NM_001105077.3, Supplementary Figure S7A). These regions adjacent to *MECOM* were fused to the *GATA2* locus at 3q21.3 (n = 2), the *CDK6* locus at 7q21.2 (n = 3), and an intergenic region at 2p21 (n = 1). Although no reads of chimeric transcripts were present in WTS, high expression of *MECOM* was observed in all six cases (Supplementary Figure S7B), which aligns with the mechanism of enhancer hijacking previously described (9). Similarly, in SJ030410, WGS identified a rearrangement juxtaposing the *HOXA* locus on 7p15 with the enhancer RNA *CDK6*-AS1 (10) on 7p21.2, and high expression of *HOXA13* was observed in WTS analysis (Supplementary Figure S7 C-D). Notably, *MECOM*-r cases associated with *CDK6* showed fusion junctions in intron 2 or 3 of *CDK6* (Supplementary Figure S7E), suggesting these leukemias are utilizing a different enhancer within the *CDK6* locus.

SJ030153 and SJ030431, classified as FAB-M0 and M1, respectively, showed a translocation t(5;14) (q35;q32) by WGS (Supplementary Figure S8A). As 14q32 rearrangements deregulating *BCL11B* have recently been recognized as a distinct molecular subtype of T-/myeloid-MPAL and myeloid immature acute leukemia (11, 12), we evaluated the expression of *BCL11B*. In both cases, *BCL11B* expression was comparable to non-*BCL11B* AML cases and dramatically lower than that in the *BCL11B*-rearranged AML subtype. Given that genomic profiling revealed characteristics similar to T-ALL with t(5;14)(q35;q32) in both cases (13, 14, 15), including alterations in *PHF6* and focal deletions of *CDKN2A/B*, we also evaluated the expression of *TLX3* and *NKX2–5*. However, neither *TLX3* nor NKX2–5 showed overexpression in either case by WTS, disagreeing with the enhancer hijacking mechanism initially predicted for both cases (Supplementary Figure S8 B-D). Together, the different case scenarios described above highlight the need for an iWGS-WTS approach in the diagnosis of enhancer hijacking structural alterations.

#### Expression data from WTS improves interpretation and molecular classification of variants

Further on, combined analyses of sequence variants, expression of involved genes (or alleles) and global gene expression profiling through WTS can provide critical insights into the biology and clinical significance of findings, which is particularly valuable when a novel and intriguing genetic alteration is identified. One example from our cohort is a *HSPA8::PRDM16* fusion identified in SJ031206. This fusion was considered biologically similar to other *PRDM16* rearrangements known to occur in AML (16, 17) because: 1) the *PRDM16* exons (exons 2–17) preserved in the fusion are the same as those in previously reported *PRDM16* fusions; 2) overexpression of *PRDM16* was observed in WTS; and 3) the global gene expression profile is compatible with the *PRDM16/MECOM-*r AML subtype (18) (Supplementary Figure S9).

Another case highlighting the value of integrating expression data obtained from WTS is SJ032178, where WGS revealed an acquired frameshift variant in the N-terminal transactivation domain of *CEBPA* at a VAF of 35% in genomic DNA. The same variant was detected by WTS at VAF of 97%, indicating exclusive expression of the mutant allele in the tumor. Furthermore, the global gene expression profile demonstrated the characteristic expression profile of AML with *CEBPA* mutations (Supplementary Figure S10A). Together, the overall findings from WTS provide a strong rationale to diagnose this patient’s leukemia as AML with *CEBPA* mutation.

As global gene expression profiling has been shown to offer additional insights into AML subtyping (7), this analysis was incorporated into our routine workflow for WTS. In this study cohort, seven cases harbored in-frame fusions involving 5’ exons of *RUNX1* fused to partner genes other than *RUNX1T1*. Global gene expression profiling clustered three *RUNX1::CBFA2T3/2* fusion cases with the *RUNX1::RUNX1T1* group, which is in concordance with other studies considering *RUNX1::CBAF2T3/2* as a form of *RUNX1::RUNX1T1*-like AML (19). In contrast, four cases with *RUNX1* fused to other genes (*EVX1*, *POU2F2*, *USP42*, and *ZEB2*) neither cluster with the *RUNX1::RUNX1T1* group nor form a distinct cluster in gene expression profiling (Supplementary figure S10B), suggesting that different biologic mechanisms may be associated with these rare *RUNX1* fusions in AML, and their clinical significance remains unclear. Overall, these case scenarios highlight the value of implementing global gene expression profiling in tumor molecular diagnostics.

### Comparing the iWGS-WTS approach to Conventional Cytogenetics

#### Detection of driver gene fusions

**Figure 3:**
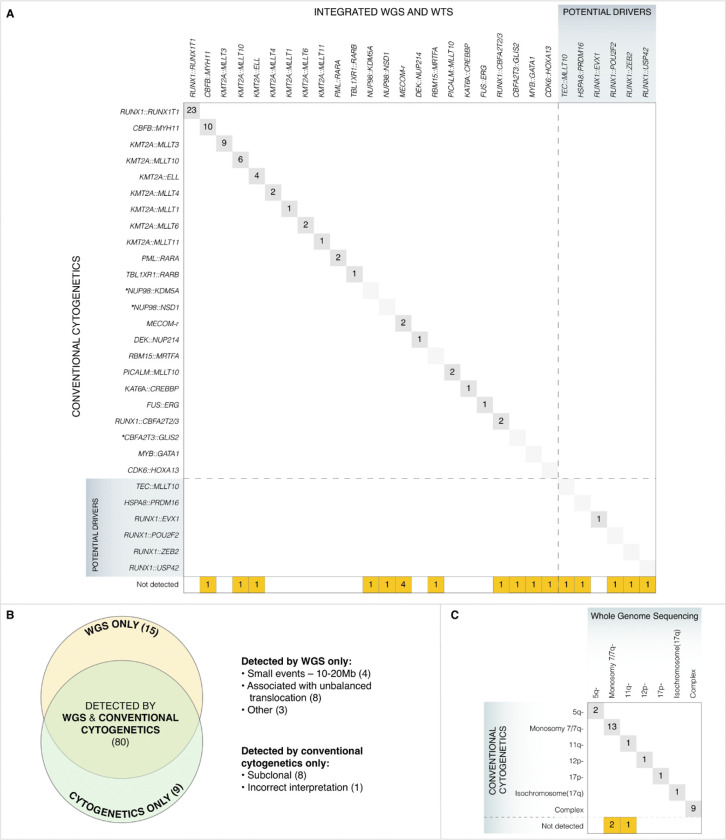
Detection of chromosomal abnormalities and comparison to conventional cytogenetics. **A)** Comparison of AML-associated fusion detection between conventional G-banded Karyotyping (with complementary FISH test when available) on the y-axis and integrated WGS and WTS (iWGS-WTS) on the x-axis. *Known cytogenetically cryptic gene fusions. **Note:** FISH (Fluorescence in situ hybridization) analysis for *KMT2A* rearrangement, *CBFB* rearrangement and *RUNX1::RUNX1T1*fusion were available in a subset of cases (See Supplementary Table S1). **B)** Overview of the comparison of WGS and conventional cytogenetics in the detection of large-scale CNVs. Groups of events detected by cytogenetics or WGS alone are listed to each side. **C)** Comparison of the detection of AML-MR defining cytogenetic alterations by conventional cytogenetics (y-axis) and WGS (x-axis).

We next compared results from iWGS-WTS with G-banded karyotyping for the detection of chromosome translocations/gene fusions in 136 cases tested with conventional cytogenetics. Among these cases, 90 had known or potential AML-driver gene fusions established by iWGS-WTS, of which 71 (~ 79%) were detected by cytogenetics with or without FISH assistance (Fig. 3A), including 6 of 7 *KMT2A::MLLT10* cases with complex or cryptic chromosome alterations for which fusions were defined using *KMT2A* break-apart FISH analysis. Discordant findings consisted mainly of rare AML fusions discovered in recent years by advanced sequencing technologies and known to be cytogenetically cryptic, for instance, *CBFA2T3::GLIS2* and *NUP98*-fusions (Supplementary Table S5). Interestingly, three cases with traditional AML-defining gene fusions that have routinely been evaluated by cytogenetic analysis for decades, were only identified by iWGS-WTS, including *CBFB::MYH11* (SJ032253), *RBM15::MRTFA* (SJ030209), and *KMT2A::MLLT10* (SJ030361). Break-apart FISH assays for *CBFB* and *KMT2A* were performed on SJ032253 and SJ030361, respectively, but did not show diagnostic evidence of gene rearrangements, suggesting that a cryptic insertion mechanism may underlie the formation of gene fusions in both cases. SJ032253 and SJ030209 were further confirmed by RT-PCR; whereas, SJ030361 had a rare fusion transcript between *KMT2A* exon 6 and *MLLT10* exon 15, for which our clinical RT-PCR assays lack a specific primer to verify. None of the cytogenetically positive AML-associated chromosome rearrangements were missed by iWGS-WTS.

#### Solving complex genomes and identifying drivers in cases with complex karyotype

We also assessed the ability of iWGS-WTS to resolve complex genomes and uncover critical pathogenic alterations in a series of 9 cases where conventional cytogenetics showed complex karyotype without further diagnostic information. Indeed, iWGS-WTS identified AML-defining genetic alterations in 7 of these 9 cases (1 *KMT2A::ELL*, 1 *RBM15::MRTFA*, 1 *NUP98::KDM5A*, 1 *NPM1* mutation, and biallelic alterations of *TP53* in 3 cases), and revealed a *CDK6::HOXA*10 and a *TEC::MLLT10* (rarely reported in-frame fusion) in the remaining two cases, SJ030410 and SJ030773, respectively (Supplementary Table S6B). Additionally, although genome-wide findings from WGS and conventional cytogenetics were largely consistent, WGS illustrated the genomic complexity with granular detail and an accuracy superior to karyotype analysis. See example cases in Supplementary Fig. 11.

#### Detection of Large-Scale CNVs

We next evaluated the concordance between WGS and conventional cytogenetics in detecting LS-CNVs that are theoretically visible by cytogenetics at a band level of 400 bands per haploid (bphs). This banding resolution allows discrimination of copy-number changes of ~ 9 Mb or higher (20). Of the 136 cases for which karyotyping was performed, 126 with non-complex karyotype were used for a head-to-head comparison study.

The overall agreement in LS-CNV findings between WGS and conventional cytogenetics was ~ 77% (80/104) (Fig. 3B and Supplementary Table S6A), including abnormal chromosomes described as unbalanced structural alterations (“add” or “del”) or marker chromosome (“mar”) by conventional cytogenetics and further clarified by WGS in 5 cases (SJ031527, SJ031601, SJ031719, SJ032364 and SJ032526). The chromosome abnormalities of these 5 cases are described in Supplementary Table S6A (see Comments) and illustrated in CIRCOS plots which integrated the visualization of genome-wide SVs and CNVs revealed by WGS (Supplementary Figure S12 A-E).

Both testing platforms detected certain aberrations that were not identified by the other (nine by conventional cytogenetics alone and 15 solely by WGS). Eight of the nine CNVs identified only by conventional cytogenetics were subclonal findings observed in < 50% of analyzed metaphases, and none defines AML, myelodysplasia-related (AML-MR) (5). The remaining CNV, a trisomy 17 in SJ030079, detected by G-banded karyotyping in poor-quality metaphases could not be confirmed by either WGS or FISH.

Among the 15 events identified by WGS only, four were relatively small CNVs (10 ~ 20Mb) which could be challenging to detect using conventional cancer cytogenetics due to the quality of the metaphases and/or G-banding resolution; the remaining were mainly CNVs associated with unbalanced chromosomal translocations which were missed by conventional cytogenetics due to banding similarity of the chromosome segments involved. It should be noted that the agreement between WGS and conventional cytogenetics to call AML-MR cytogenetic abnormalities (5) throughout the cohort was ~ 90% (28/31) (Fig. 3C). All 3 discordant findings were segmental deletions missed by conventional cytogenetics, including 2 cases with deletions in 7q and one deletion in 11q.

### Molecular diagnosis and classification through integrated genomic and transcriptomic profiling

#### Molecular subtypes defined by integrated genomic and transcriptomic profiling

**Figure 4:**
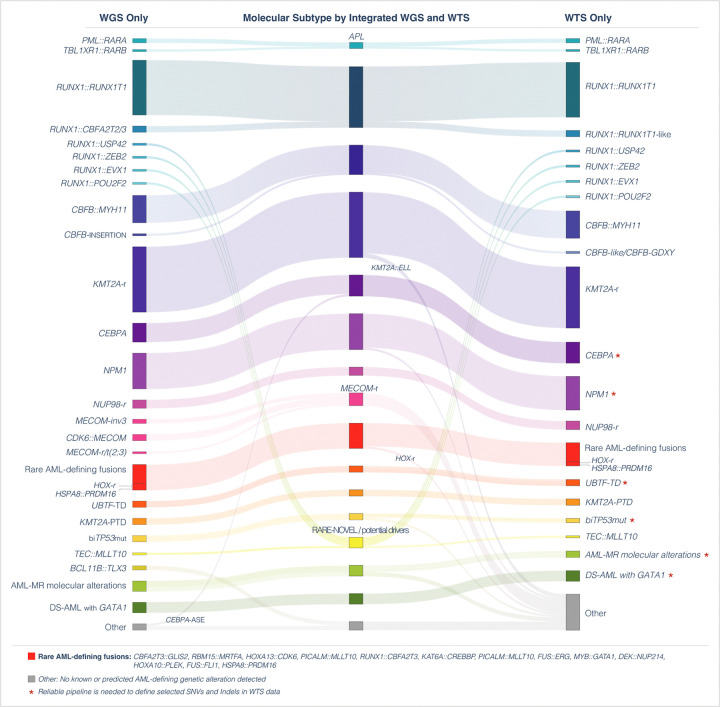
Molecular classification of cases in the study cohort. Comparison between different tumor profiling approaches: WGS, WTS, and integrated WGS and WTS (iWGS-WTS), for the molecular classification of AML. Sankey diagram shows detection of AML genetic drivers by WGS only on the left, WTS only on the right and the iWGS-WTS in the middle.

The molecular subtype of each case was determined based on the comprehensive and real-time genomic and transcriptomic profile by iWGS-WTS. When comparing the molecular classification by WGS alone, WTS alone and iWGS-WTS, we observed the highest diagnostic detection rate and precision with iWGS-WTS (Fig. 4). Of the 149 non-Down syndrome patients, 91 were characterized by AML-class defining gene fusions (5, 21), 30 by AML class-defining mutations (5, 21), 13 defined by newly proposed AML class-defining variants (7, 17, 18, 22–24), and five cases showed AML-MR defining gene mutations and/or cytogenetic abnormalities in the absence of any other known or potential genetic drivers of AML. Of the other 10 cases, novel or rare in-frame gene fusions previously reported in hematological malignancy case reports in literature (25–27) were identified in six, while the genetic driver for the last four cases remained unclear. Pathogenic *GATA1* variants were identified in all five patients with Down syndrome.

#### Additional pathogenic/likely pathogenic genetic aberrations co-occurring with driver genetic alterations

**Figure 5:**
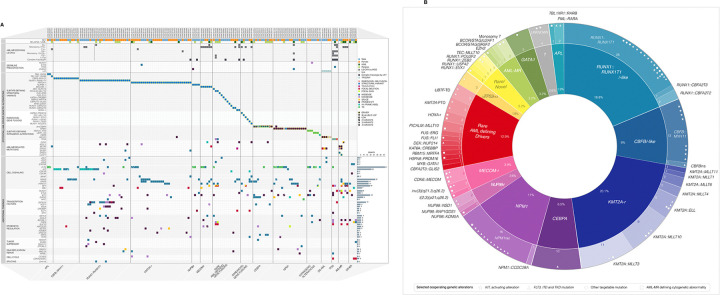
Genomic landscape of cases in the study cohort. **A)** Pathogenic/likely pathogenic genomic alterations identified in 154 AML cases by the integrated WGS and WTS approach (Note: SJ032376 sequenced by WES and WTS). Patients’ sex, treatment-related AML (tAML) and relapsed cases are shown on the top. Only alterations seen in two or more cases in this study cohort are presented for additional findings, total count is shown on the right. Molecular subtypes are indicated along the bottom. **B)** The donut chart breaks down the 154 AML cases into subtypes according to genetic drivers/potential drivers determined by the integrated WGS and WTS approach. Absolute count is listed as numbers on the outer ring, and the percentage of the total cohort is shown towards the inner ring. Selected additional genetic alterations, either with impact on risk assessment and/or being directly targetable/therapy-informing, were shown and specified by different symbols along with the driver alteration. AML-MR, acute myeloid leukemia - myelodysplasia related; APL, acute promyelocytic leukemia; *CEBPA*-ASE, *CEBPA*-allele specific expression; DS-AML, Down Syndrome-associated AML; LS-CNV, large-scale copy cumber variant; LOF, loss of function; CN-LOH, copy neutral loss of heterozygosity; N/A, not available; PTD, partial tandem duplication; tAML, therapy-related AML; TD, tandem duplication.

Along with the detection of major genetic driver alterations, comprehensive genomic profiling uncovered additional genetic abnormalities (Fig. 5A, Supplementary Tables S2-S5), allowing for a comprehensive picture to draw conclusions for prognosis/risk stratification.

Among all 154 patients, 430 additional P/LP alterations (SNVs/indels, focal CNVs, FLT3-ITDs, non-driver SVs, adverse LS-CNVs) were detected in 141 (92%) patients, at an average of 3 per patient. Approximately 25% of these additional findings were of direct clinical relevance, impacting risk assessment and/or being directly targetable or therapy-informing (Fig. 5B). For example, patient SJ032210, an *NPM1*-AML case, harbored deletions in 5q and 13q, which are associated with a poorer prognosis (28, 29). Another example, iWGS-WTS identified activating alterations in *KIT* in 17 of 39 core-binding factor AML cases in this study. Although only exon 17 mutations in *KIT* have been established as a poor prognostic factor in *RUNX1::RUNX1T1*-AML (30), other *KIT* activating mutations may also serve as potential molecular targets for AML treatment, particularly in relapsed cases (31).

### Germline origin genetic alterations

Among the 220 genes predisposing to cancer and/or bone marrow failure that are clinically validated for germline molecular diagnosis at SJCRH (Supplementary Table S7A), 22 P/LP variants were identified in 20 patients (~ 13%) (Supplementary Table S7B). Heterozygous P/LP germline alterations in genes known to predispose to hematologic neoplasms with autosomal dominant inheritance were identified in six patients, including variants in *CHEK2* (seen in 2 patients), *ETV6*, *RUNX1* and *TP53*, as well as intragenic deletions in *PALB2* and *UBE2T*. Patient SJ030778 had one pathogenic variant in *RUNX1* and an intragenic deletion in *PALB2*. Two of these six patients had therapy-related AML, including one patient (SJ030708; *TP53*) previously treated for Ewing sarcoma and the other patient (SJ032210; *CHEK2*) with a history of neuroblastoma. In addition to these six patients, patient SJ030509 had biallelic pathogenic variants in *SBDS*, and six patients were heterozygous for P/LP variants in genes with autosomal recessive inheritance that are known to predispose to hematopoietic malignancy. Incidental findings of P/LP germline variants in genes associated with non-hematologic malignancies were seen in seven patients, including heterozygous *MUTYH* G396D identified in four patients, *MITF* E419K in two, and a *CDH1* loss-of-function in one patient.

## Discussion

Integrating WGS into cancer molecular diagnosis offers several advantages to standard diagnostics, including detection of non-coding variants and greater sensitivity for identifying copy number and structural variants (32). Emerging studies also highlighted the potential of transcriptomic analyses to provide accurate and comprehensive diagnostic information for AML and other cancers, including molecular subtyping, risk assessment, and targeted therapy (33, 34). Our institution and others have demonstrated that combining WGS, WES, and WTS significantly enhances tumor diagnosis and clinical care (35, 36, 37). While WGS shows great promise as a comprehensive diagnostic tool, its successful adoption requires addressing technical concerns to ensure accurate and timely leukemia diagnostics.

In this study, we reviewed real-time clinical testing results from the iWGS-WTS test in 154 pediatric AML patients and compared them with targeted NGS, and conventional molecular and cytogenetic tests, to assess the detectability, accuracy, and clinical utility of iWGS-WTS in identifying a wide range of genomic variants. Fifindings were reported independently, allowing for a robust and unbiased comparison of methods. This comprehensive analysis provides strong evidence supporting the use of iWGS-WTS approach to effectively identify key genetic drivers and cooperating genetic lesions for precise disease classification and risk assessment in pediatric AML.

Our study demonstrated that iWGS-WTS is comparable to WES and high sequencing-depth panel assays in detecting clinically relevant SNVs/indels. Specifically, iWGS-WTS identified all variants with a VAF > 8% and 94.5% of variants with a VAF ≥ 5% detected by NGS panel testing, despite ~ 20-fold lower sequencing coverage than targeted panel sequencing. Unlike targeted NGS testing, WGS and WTS enable rapid clinical adoption of new diagnostic or prognostic markers through updates to bioinformatics pipelines and reporting, without the need for laborious and resource-consuming assay development. For example, we detected 3 cases of AML with *UBTF* tandem duplications, a newly reported driver alteration (23) that is not present on most commercial panels. Additionally, previously generated WGS and WTS data can be reanalyzed for emerging biomarkers when clinically necessary or for translational research. This capability is critical in molecular oncology, given the rapid pace of cancer research, the continuous discovery of new markers, and the typically limited availability of patient diagnostic samples.

Classical chromosome banding analysis for assessing numerical and structural chromosomal abnormalities has traditionally played an essential role in the diagnosis and prognostication of AML. However, chromosomal rearrangements that are detectable by conventional cytogenetic analysis are restricted to large abnormalities with microscopically visible banding differences; cryptic and complex rearrangements could be missed or misinterpreted. Both WGS and WTS allow for the accurate and robust detection of such gene fusions, regardless of the complexity of the tumor genome. In this study, 18 AML-driver gene fusions identified by iWGS-WTS were missed by conventional karyotyping due to their cryptic nature or masked within complex cytogenetic alterations.

The comparison of LS-CNV detection between conventional cytogenetics and WGS demonstrated that WGS not only identified all AML-MR defining cytogenetic abnormalities but also exhibited superior capability in illustrating the composition of abnormal chromosomes and resolving complex genomes which often make G-banded karyotype analysis time-consuming, subjective, and prone to errors. Although WGS could miss sub-clonal large gains or losses with a clone size below 30%~40% if no SV is associated with the CNV event (example: whole chromosome or chromosome arm gains or losses), the precise resolution of the SV breakpoints in WGS analysis (38) gives greater confidence in identifying segmental CNVs. Our real-time clinical testing workflow, which combines traditional CNV analysis algorithms with soft clip reads at both boundaries of suspected CNVs, identified 12 small CNVs (< 10 kb) in this cohort that affect genes frequently involved in AML. In contrast, genome-wide SNP-array diagnostic assays often use filters requiring at least 20 consecutive markers in a region of ~ 50 kb (39). Consequently, clinically relevant cryptic CNVs could be missed by SNP-array assays.

Although WGS is capable of providing a complete spectrum and unbiased view of genomic abnormalities involved in cancer development, we demonstrated the need for iWGS-WTS to improve the molecular diagnosis and classification of AML. Additional information derived from WTS, including the presence or absence of in-frame fusion transcripts, ectopic expression of oncogenes, allele-specific expression, and interrogating global gene expression signatures, could further influence decision making. On the other hand, although WTS also contributed significantly to the identification of the full spectrum of genetic variants, its application depends on the presence of genomic alterations in the transcriptome and, as a consequence, events that lead to deregulated gene expression but no fusion transcripts (e.g. enhancer hijacking) are challenging to detect with this approach; also nonsense mutations and frameshift indels may not be detectable by WTS due to nonsense-mediated mRNA decay.

**Figure 6:**
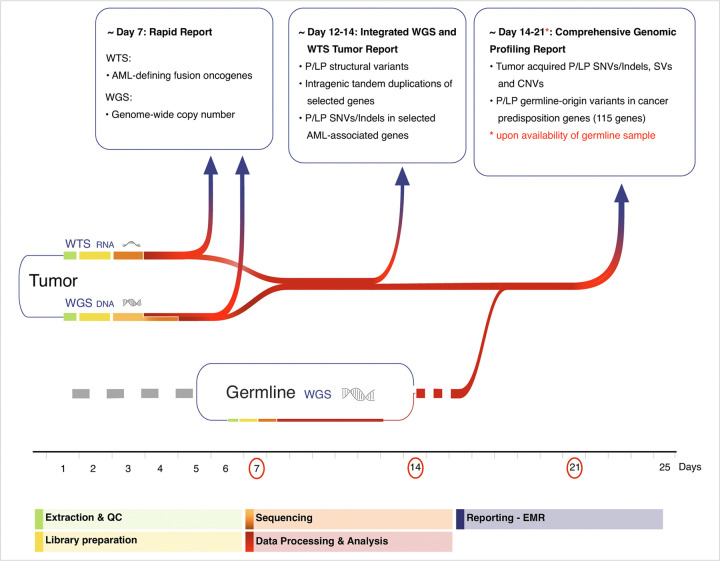
Advanced clinical workflow for the integrated WGS and WTS testing. A phased data processing and reporting approach for WGS and WTS is shown with timeline indicated below. Segments in different colors indicate varying steps of the process from sample acquisition/processing to data processing and analysis. Reports can be released in three phases, as indicated above: rapid tumor report by day 7, selected integrated iWGS-WTS tumor report by day 14, and a comprehensive paired tumor-normal genomic profiling report by day 21. EMR, electronic medical record; P/LP, pathogenic/likely pathogenic; SNV, single nucleotide variant; SV, structural variant; QC, quality control.

As a diagnostic test, turnaround time (TAT) of the iWGS-WTS is a critical factor to consider for clinical decision-making. Traditional diagnostic tests for AML typically have a TAT of 7–14 days. With increased awareness of germline predisposition to cancers, the identification of germline P/LP variants is essential for accurate diagnosis, patient management, and screening of related donors for stem cell transplantation (35, 40). Hence, when considering the TAT of iWGS-WTS, it is important to balance between a fast diagnosis of the tumor in a targeted manner and comprehensive tumor/germline profiling. Throughout the duration of this study, we developed a phased approach, allowing early identification and reporting of key genetic alterations with significant diagnostic or prognostic implications in tumor sample, while concurrently performing paired tumor-normal analysis for a comprehensive somatic and germline report. The workflow, outlining both current practices and components under implementation, is illustrated in Fig. 6. This phased data process and reporting approach allows most AML-defining genetic drivers and genome-wide LS-CNVs to be reported within seven days; while rare/novel but potentially oncogenic gene fusions and select cooperating variants could be reported by day 14. Upon availability of a matched germline sample, a full report, including a complete tumor genomic profile and germline findings for cancer predisposition, could be issued within 2–3 weeks.

The diagnosis of AML has traditionally relied on multiple tests, each requiring different sample collection, equipment, reagents, and skills of conducting and interpreting individual tests, to collectively enable leukemia classification and risk stratification. The advancement of WGS and WTS, especially our successful implementation of iWGS-WTS in the routine molecular diagnosis of pediatric AML, has demonstrated the potential to improve resource allocation, increase the speed of accurate disease classification and management plan development, and consequently reduce costs for the care of AML patients. An added benefit is the potential to use this comprehensive genomic information for future research discoveries, including our efforts to share it with the global research community through St. Jude Cloud (https://www.stjude.cloud/). Our recent and ongoing improvement of workflows indicate that the TAT of the iWGS-WTS could be comparable to or even better than the traditional/standard tests. We believe that this study provides strong evidence and a solid rationale for reconstructing the pediatric AML diagnostic workup by replacing conventional and standard molecular diagnostic approaches with iWGS-WTS. This study may also pave the way for enhancing molecular diagnostics for other pediatric cancers.

## Materials and Methods

### Patient, cell lines, and testing platforms

A total of 154 pediatric and adolescent patients with AML, admitted to SJCRH (n = 136) and/or enrolled in SJCRH-sponsored clinical trials (n = 18), were included in the study cohort between 2016 and 2021. Informed consent was obtained from all patients for clinical genomics and research. Tumor and matched germline samples were collected from each patient for paired tumor-normal clinical genomics testing in our Clinical Laboratory Improvement Amendments (CLIA)-certified Molecular Pathology clinical laboratory. Bone marrow aspirate or peripheral blood samples with documented blast counts (see Supplementary Table S1) were used as tumor samples. Skin biopsy samples were collected as germline comparators, other tumor-free samples (see Supplementary Table S1) were used for patients when skin biopsy samples were not available. All except one patient received real-time diagnostic genomic profiling of their tumor samples by WGS and WTS; the remaining tumor sample was tested only by WES and WTS due to limited DNA yield from extraction, which was inadequate for WGS. In addition, WES was performed for all cases in conjunction with WGS for the internal assessment of the detection of SNVs and indels by WGS. Material from 136 of 154 patients was subjected to conventional cytogenetics evaluation in the Clinical Cytogenetics laboratory at SJCRH as part of the traditional diagnostic workup. Additionally, a tumor cell line, COLO 829 (CRL-1974, ATCC, USA), and its matched normal cell line, COLO 829BL (CRL-1980, ATCC, USA), were used to estimate the WGS testing performance for SNVs, small indels, and large-scale CNVs.

### WGS, WES, and WTS library preparation and sequencing

DNA was directly extracted from fresh samples or mononuclear cells that were purified from bone marrow or peripheral blood by density gradient centrifugation (Ficoll) using phenol-chloroform or Chemagic 360 auto-extraction (Revvity chemagen Technologie GmbH, USA). RNA was extracted using TRIzol reagent (Thermo Fisher Scientific, USA) or by Chemagic 360 auto-extraction. DNA and RNA integrity was assessed by agarose gel electrophoresis and Agilent 2100 BioAnalyzer (Agilent Technologies, USA), respectively. DNA and RNA concentrations were measured using a Qubit Fluorometer (Thermo Fisher Scientific, USA). The WGS, WES, and WTS tests were performed as previously described (41). In brief, high molecular weight DNA was extracted, and 1μg and 150ng DNA from each sample was used for WGS and WES, respectively. The minimum total RNA input for WTS library preparation was 250ng. Sequencing libraries were constructed using the TruSeq DNA PCR-Free sample preparation kit (Illumina, Inc., USA) for WGS, the TruSeq exome enrichment kit v1 (Illumina, Inc., USA) for WES, and the TruSeq Stranded Total RNA Kit (Illumina, Inc., USA) for WTS. All sequencing was done with HiSeq4000 (2017- early 2020) or NovaSeq 6000 (2020 and onward).

### Sequencing data analysis, variant calling and classification

Sequencing data were processed by our computational bioinformatics pipeline as previously described (41). The variant output from this pipeline was further processed following our auto-calling rules (Supplementary Figure S1) and valid alterations were subjected to variant classification. The pathogenicity of both somatic and germline variants was assessed according to guidelines put forth by the Association for Molecular Pathology (AMP) and the American College of Medical Genetics and Genomics (ACMG) guidelines, public database, published literature, and internal experience (42–46).

### Targeted DNA sequencing

Results from targeted NGS (Archer^™^ VARIANT*Plex*^™^ Myeloid panel, Integrated DNA Technologies, USA) obtained from the same samples used for WGS, WES, and WTS, were available for 39 AML patients. The sequencing library was prepared with 50–100ng DNA input and sequenced on MiSeqDx (Illumina, USA). Sequencing data were analyzed by Archer^®^ Analysis software (version 6.2) and variant calling was determined based on the default criteria provided by the vendor. This targeted NGS panel covers 75 genes and can detect SNVs and small indels at a variant allele frequency (VAF) as low as 3%.

### Conventional cytogenetic analysis

Conventional cytogenetic analysis was performed on 24-hour unstimulated bone marrow cultures with or without synchronization according to standard procedures. At least 20 metaphases were analyzed, and karyotypes were interpreted according to the International System for Human Cytogenetic Nomenclature. Fluorescence in-situ hybridization (FISH) was performed as needed.

## Data Availability

The next generation sequencing data for the current study are available from the corresponding author on reasonable request.
